# Intestinal dysbacteriosis induces changes of T lymphocyte subpopulations in Peyer’s patches of mice and orients the immune response towards humoral immunity

**DOI:** 10.1186/1757-4749-4-19

**Published:** 2012-12-11

**Authors:** Fei Gao, Ming Li, Yinhui Liu, Chuanzhou Gao, Shu Wen, Li Tang

**Affiliations:** 1 Department of Microecology, Dalian Medical University, No.9 Western Section, Lvshun South Street, Lvshunkou District, Dalian, 116044, China; 2 Central Laboratory of Dalian Medical University, Dalian Medical University, No.9 Western Section, Lvshun South Street, Lvshunkou District, Dalian, 116044, China

**Keywords:** Intestinal dysbacteriosis, Peyer’s patches, Immune response, T lymphocytes, Cytokines

## Abstract

The large numbers of human intestinal microorganisms have a highly co-evolved relationship with the immune system. Dysbacteriosis of intestinal microbiota induces alterations of immune responses, and is closely related to disease development. Peyer’s patches are immune sensors in intestine which exert essential functions during development of inflammatory disease. However, interactions between commensal bacteria and PPs have been poorly characterized. In this study, changes of lymphocyte subpopulations and production of cytokines in PPs of mice with intestinal dysbacteriosis were investigated. The ceftriaxone-induced dysbacteriosis caused a notable change in populations of T lymphocytes, their subpopulations in PPs and expressions of various cytokines. Our results suggest intestinal dysbacteriosis in mice reduces immune tolerance in PPs and orients immune response towards humoral immunity.

## Background

Intestinal microorganisms are required for proper development of the immune system, as indicated by the fact that germ-free (GF) mice have poorly developed lymphoid tissues [1]. Under normal conditions, gut microflora exists in a state of equilibrium with host that is mutually beneficial to the degree that it has been described as a separate ‘organ’ adapted to human physiology [2]. However, factors such as antibiotics abuse, stress, or nutritional deficiencies can seriously disrupt this delicate balance, resulting in altered immune responses and disease. In IBD patients, the quantity of commensal bacteria in the intestine is reduced, diversity of microbiota is also altered [3,4]. Patients with IBD exhibit disruption in mucosal integrity, dysfunction of gut-associated lymphoid tissue (GALT), and deregulated immune tolerance [5,6]. Nevertheless, whether microbial dysbiosis in the gut is the cause of dysfunction in GALT, and of reduced immune tolerance, are as yet poorly documented. Peyer’s patches (PPs) are major component of GALT. Lacking of PPs and lymph nodes would develop more severe colitis in mice [7], suggesting PPs’ importance in exerting immune functions during the development of IBD. The participation of PPs in inflammatory disorders and their interplay with gut microbiota is thus becoming an interesting field of research.

### Study design and results

**Figure 1:**
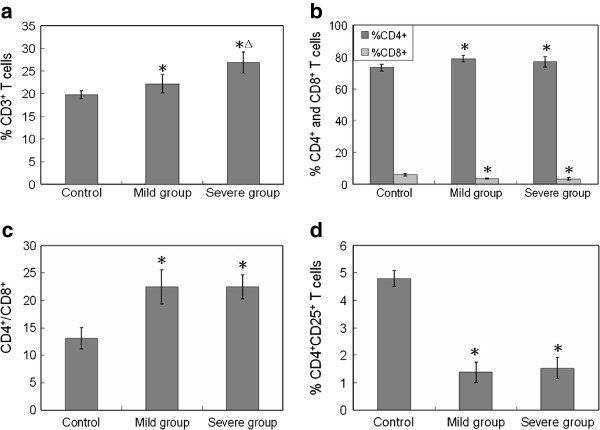
**Changing of lymphocyte population and subpopulations in PPs of different experimental groups.a**), Percentage of CD3^+^ T cells among total cells; **b**), Percentage of CD4^+^ and CD8^+^ cells among CD3^+^ T cells; **c**), The proportion of CD4^+^/CD8^+^ ; **d**), Percentage of CD4^+^CD25^+^ T cells among CD3^+^ T cells. *, p<0.05, compared with control group; Δ, p<0.05, compared with mild group. Five BALB/c mice were administered intra-gastrically with 0.2 mL of ceftriaxone sodium (200 mg/mL or 400 mg/mL) twice a day with an interval of 6 hours for 4 or 8 days to establish the mild or severe intestinal dysbacteriosis mouse models. Five BALB/c mice treated with sterile water instead of ceftriaxone sodium served as control (Details in Additional file 1: Supplementary material).

**Figure 2:**
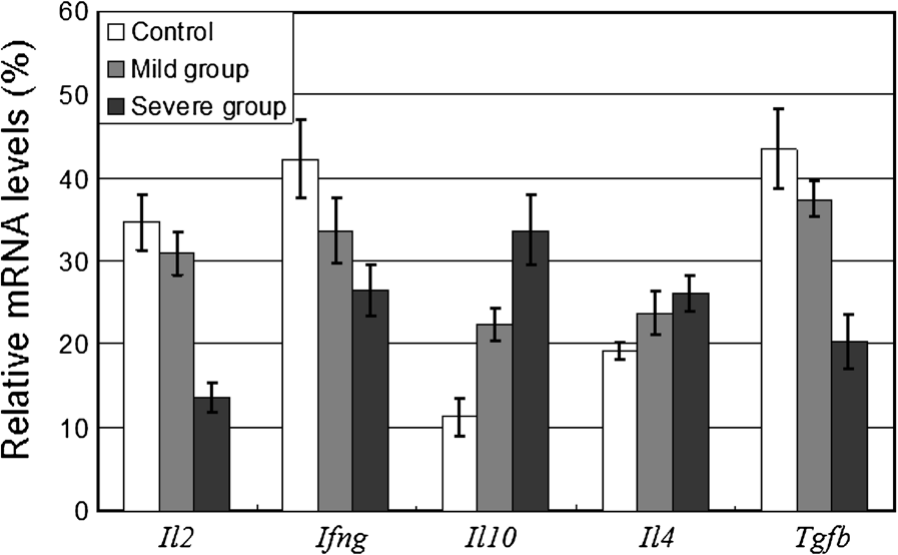
**The expression levels of mRNA for cytokines.** The relative mRNA abundance of cytokines in PPs of different experimental groups was detected by Reverse-transcript PCR (Details in Additional file 1: Supplementary material and methods). *Tgfb*, transforming growth factor-beta; *Il2*, interleukin-2; *Ifng*, interferon-gamma; *Il4*, interleukin-4; *Il10*, interleukin-10. The relative mRNA abundence is determined by dividing the intensity of cytokine PCR products with the intensity of *β*-*actin* PCR product. Values are means±SEM (n=3).

We hypothesized that alterations in gut microflora may induce changes of cellular composition and cytokine productions in PPs, and lead to a shift in immune response/tolerance. To test this, we established mouse models with dysbacteriosis through intragastric administration of ceftriaxone sodium. Alterations of lymphocytes in PPs (particularly the T lymphocyte subpopulations) were examined, as was cytokine mRNA expression (Additional file 1: Supplementary material). The population of commensal bacteria was found reduced in mice treated with ceftriaxone sodium and some are even eradicated (Additional file 2: Table S1). The structure of intestinal microflora was seriously disrupted. The population of CD3^+^ T cells, which represents the level of cell-mediated immune response, was detected by flow cytometry. Compared with control mice, the total level of CD3^+^ T cells in PPs of mild dysbacteriosis mice increased to 22.2%, and to 26.9% in PPs of the severe group (Figure 1a). The T lymphocyte subpopulations were also examined. Results in Figure 1b show that CD4^+^ T cells in PPs of mild and severe dysbacteriosis mice increased by 7.37% and 5.15%, respectively. In contrast, the CD8^+^ T cells in PPs of mice with dysbacteriosis decreased from 5.85% to no more than 3.60%, resulting in a higher ratio of CD4^+^/CD8^+^ compared with healthy mice (Figure 1c). Changes in CD4^+^/CD8^+^ ratio are normally considered as an important reference to predict the shifting of immune responses. The increased proportion of CD4^+^/CD8^+^ indicates that intestinal dysbacteriosis has induced humoral immunity in mice. The populations of CD4^+^CD25^+^ T cells in PPs of healthy and dysbacteriosis mice were also detected. CD4^+^CD25^+^ are marker molecules on the surface of regulatory T cells (Treg cells), which are crucial for maintenance of immunological tolerance [8]. In PPs of dysbacteriosis mice groups, the regulatory CD4^+^CD25^+^ T cells were severely decreased (Figure 1c). These striking decreases indicate a significant reduction of immunological tolerance. The transforming growth factor-beta (TGF-β) is a critical regulator for the development of Treg cells, which are crucial for the maintenance of immunological tolerance [9]; we therefore detected the mRNA expression levels of TGF-β gene. As shown in Figure 2, a decreasing pattern was observed in dysbacteriosis mice groups, which suggested a delay of Treg cells development. This is basically correlated with the trends that have seen for the population of CD4^+^CD25^+^ T cells. Th cells represent a functionally heterogeneous population, comprising mainly two distinct subsets (termed Th1 and Th2 cells), defined by their cytokine secretion profiles [20]. Th1 cells produce IL-2, IFN-γ and tumour necrosis factor-beta (TNF-β), and promote both macrophage activation, and the production of complement fixing and opsonising antibodies. Th2 cells, which synthesize cytokines such as IL-4, IL-10 and etc., provide optimal help for antibody production, promote mast cell growth, eosinophil differentiation and activation, resulting in humoral or allergic responses [10]. In the present study, a slight decrease (by 10.7%) of *Il2* gene expression was observed in PPs of mice with mild dysbacteriosis, while a dramatic decrease (by 60.9%) was observed in the severe group (Figure 2). The mRNA levels of the *Ifng* gene also decreased (by 20.4% and 37.4%, respectively). Expression of the *Il4* gene in PPs of mice with mild dysbacteriosis increased by 23.6% compared with control, and it was 36.4% higher in the severe group. Expression of the *Il10* gene increased dramatically in PPs of both mild (by 98.9%) and severe (by 200.4%) groups. The decrease in mRNA levels of *Il2* and *Ifng* in PPs of dysbacteriosis mice suggest activation and proliferation of Th2 cells, while the elevation of *Il4* and *Il10* inhibit Th1 cells. Results in Figure 2 thus support the observation that dysbacteriosis in mice has induced a shifting of immune responses in PPs towards humoral immunity.

## Conclusions

The present work describes the effects of intestinal dysbacteriosis on immune responses of PPs in mice. This disturbance in mice can be induced by ceftriaxone sodium, making it an ideal animal model to mimic gut dysbacteriosis in human. Also, this is the first study to document changes in T lymphocyte subpopulations and cytokine mRNA expression of PPs in response to intestinal dysbacteriosis. Dysbacteriosis induced a notable change in overall population of T lymphocytes, and in T lymphocyte subpopulations, in PPs, as well as altering the mRNA expression of various cytokines. These data suggest that immune tolerance in PPs is reduced after the administration of antibiotics, and the immune response has shifted to humoral immunity. However, relevance of these observations requires further study.

## Competing interests

The authors declare that they have no competing interests.

## Authors’ contributions

LT and SW designed the research; FG and ML performed the experiments; ML and YL analyzed the data; ML, CG, SW and LT wrote this paper. FG and ML contributed equally to this work. All authors read and approved the final manuscript.

## Supplementary Material

Additional file 1 Supplementary material and methods. Click here for file

Additional file 2 **Table S1.** The population of commensal bacteria in cecal contents of mice. Click here for file

## References

[B1] Eckburg PB Bik EM Bernstein CN Purdom E Dethlefsen L Sargent M Gill SR Nelson KE Relman DA Diversity of the human intestinal microbial flora Science 2005 308 1635 1638 10.1126/science.1110591 15831718 PMC1395357

[B2] Frank DN St Amand AL Feldman RA Boedeker EC Harpaz N Pace NR Molecular-phylogenetic characterization of microbial community imbalances in human inflammatory bowel diseases Proc Natl Acad Sci 2007 104 13780 13785 10.1073/pnas.0706625104 17699621 PMC1959459

[B3] Manichanh C Rigottier-Gois L Bonnaud E Gloux K Pelletier E Frangeul L Nalin R Jarrin C Chardon P Marteau P Roca J Dore J Reduced diversity of faecal microbiota in Crohn's disease revealed by a metagenomic approach Gut 2006 55 205 211 10.1136/gut.2005.073817 16188921 PMC1856500

[B4] Ott SJ Musfeldt M Wenderoth DF Hampe J Brant O Folsch UR Timmis KN Schreiber S Reduction in diversity of the colonic mucosa associated bacterial microflora in patients with active inflammatory bowel disease Gut 2004 53 685 693 10.1136/gut.2003.025403 15082587 PMC1774050

[B5] Chandran P Satthaporn S Robins A Eremin O Inflammatory bowel disease: dysfunction of GALT and gut bacterial flora (I) Surgeon 2003 1 63 75 10.1016/S1479-666X(03)80118-X 15573623

[B6] Chandran P Satthaporn S Robins A Eremin O Inflammatory bowel disease: dysfunction of GALT and gut bacterial flora (II) Surgeon 2003 1 125 136 10.1016/S1479-666X(03)80091-4 15570747

[B7] Spahn TW Herbst H Rennert PD Lugering N Maaser C Kraft M Fontana A Weiner HL Domschke W Kucharzik T Induction of colitis in mice deficient of Peyer's patches and mesenteric lymph nodes is associated with increased disease severity and formation of colonic lymphoid patches Am J Pathol 2002 161 2273 2282 10.1016/S0002-9440(10)64503-8 12466141 PMC1850913

[B8] Watanabe N Wang YH Lee HK Ito T Cao W Liu YJ Hassall's Corpuscles instruct dendritic cells to induce CD4+CD25+ regulatory T cells in human thymus Nature 2005 436 1181 1185 10.1038/nature03886 16121185

[B9] Atarashi K Tanoue T Shima T Imaoka A Kuwahara T Momose Y Cheng G Yamasaki S Saito T Ohba Y Taniguchi T Takeda K Hori S Ivanov II Umesaki Y Itoh K Honda K Induction of colonic regulatory T cells by indigenous clostridium species Science 2011 331 6015 337 341 10.1126/science.1198469 21205640 PMC3969237

[B10] Jiang H Chess L An integrated view of suppressor T cell subsets in immunoregulation J Clin Invest 2004 114 1198 1208 15520848 10.1172/JCI23411 PMC524238

