# Supplementary material for: Intestinal dysbacteriosis induces changes of T lymphocyte subpopulations in Peyer’s patches of mice and orients the immune response towards humoral immunity
Source: Gut Pathog. 2012 Dec 11;4:19. doi: 10.1186/1757-4749-4-19 (PMC3583793; doi:10.1186/1757-4749-4-19)
Supplement: Additional file 1 — Supplementary material and methods. [file 1757-4749-4-19-S1.doc]

# Supplementary material and methods

## Mouse model of intestinal dysbacteriosis induced by Ceftriaxone sodium

Inbred BALB/c mice (aged 6-8 weeks, weighing 18 ± 22 g) were provided by the Experimental Animal House of Dalian Medical University, where they were maintained under SPF conditions. Ceftriaxone sodium was purchased from Zhongnuo Pharmaceutical Ltd., Hebei, China. To induce intestinal dysbacteriosis, 5 BALB/c mice (for each group, 3 male and 2 female mice were used) were administered intra-gastrically with 0.2 mL of ceftriaxone sodium (200 mg/mL or 400 mg/mL) twice a day with an interval of 6 hours for 4 or 8 days to establish the mild or severe intestinal dysbacteriosis mouse models. 5 mice treated with sterile water instead of ceftriaxone sodium served as the control.

## Determination of intestinal microflora

Samples from terminal ileum contents were collected in sterile tubes, weighed, and then transferred into fresh sterile tubes containing the appropriate anaerobic buffer. The samples were then diluted 10-fold and cultured for the presence of bacteria on selective agar media: MRS with vancomycin and bromocresol green medium for *Lactobacillus* selection; Blaird-paker agar medium for *Staphylococcus* selection; Rose Bengal Medium for the selection of yeasts; *Bifidobacterium* selective agar (BS); eosin methylene blue agar for *Enterobacter* selection; *Bacteroid* selective agar (BDS) medium; *Enterococcus* selective agar (EC); TATAC medium for *Streptococcus* selection; VS for *Veillonella* selection; PMS for the selection of *Peptococcus*; FS for the selection of *Fusobacterium*; CS for the selection of Eubacterium and peptone glucose agar (PGA) for Aspergillus. All the media were purchased from Hangzhou Microbiological Company, Hangzhou, China. The agar pates were incubated at 37°C for 48 or 72 hours under anaerobic or aerobic conditions, respectively. At the defined times, CFUs on each plate were counted and the number of bacteria in each sample were determined based on the original weight of the sample at the time of collection.

## Cell isolation from Peyer’s patches and Flow cytometry detection

Mice were euthanized by cervical dislocation and soaked in 75% alcohol for 3~5 min, after which the small intestine was removed. PPs were cut out and washed 2~3 times with D-Hank’s solution, and were mechanically disrupted in 5 mL 0.02 mM EDTA for 20 min at 250 r.p.m. After filtration, the PPs were washed again 2~3 times with D-Hank’s solution, and then treated with 5 mL 1.5 mg/mL IV collagenase for 40 min at 37°C. The reaction was terminated by adding the same volume of RPMI-1640 medium. Cell suspensions thus prepared were filtered through 200 mesh nylon net. Cells were centrifuged at 1200 r.p.m. for 10 min at 4°C, washed 3 times with D-Hank’s solution, and stored on ice.

For flow cytometry analysis, cell suspensions (1×10^6^) were incubated with anti-CD3, anti-CD4, anti-CD8, or anti-CD25 monoclonal antibodies (BioLegand, U.S.A.) labelled with FITC, PE, PerCP, and PerCP, respectively. Monoclonal antibodies and cells were incubated on ice in PBS-1% bovine serum albumin (Shijiqing Medical, Hangzhou, China) for 30 min and then washed three times with PBS-1% bovine serum albumin. The cells were gently resuspended in 300 μL 4% paraformaldehyde and incubated for 15 minutes at room temperature in the dark and subjected to flow cytometric analysis within 48 hours.

## Detection of expression levels of mRNA for cytokines by semi RT-PCR

Total RNA was isolated from the Peyer’s patches using TaKaRa RNAiso reagent (TaKaRa, Dalian) according to manufacture’s instruction. The residue genomic DNA was removed by DNase digestion, following the RNA clean up, RNA concentration was analyzed by measuring A260. cDNA was synthesized by oligo dT-Adeptor primers using AMV Reverse Transcriptase (TaKaRa, Dalian), under following conditions: 50°C 20 min, 99°C 5 min, 5°C 5 min, and 4 °C forever. The PCR from each sample was carried out in a total volume of 25 μL containing 5 μL of cDNA, 5 μL of 5X PCR buffer (containing 2 mmol/L dNTPs), 0.25 μL of each forward, backward primer, 0.13 μL TaKaRa Ex Tag HS DNA polymerase and 14.37 μL water. The reaction was performed in a Thermal Cycler PCR Machine (Thermo, USA) using a 30-cycle program starting with denaturation at 94 °C for 2 min followed by a three-step temperature cycling of (94°C for 30 min, Tm of different primers listed in supplementary Table S2, and 72°C for 1 min) and then terminated by a 72°C incubation for 7 min and by cooling at 4°C. The PCR products obtained were subjected to gel electrophoresis on 1.5% agarose gels containing 1X Tris borate ethylenediamine tetraacetic acid buffer and visualized by staining with ethidium bromide using a UVP biosystem (UVP). Bands were quantiﬁed using gel analysis software (Bio-Rad Laboratories, Hercules, CA, USA). The density of each mRNA band was normalized to the expression of β-actin. Results were expressed as means and standard error of means (SEM) of three independent estimations from each group.

## Statistical analysis

The data were subjected to analysis of variance using SPSS 13.0 (SPSS Inc) and differences among the means were analyzed using T-test. Values were expressed as means ± SEM. Differences of P-value < 0.05 were considered signiﬁcant.
