# Supplementary material for: Intestinal dysbacteriosis induces changes of T lymphocyte subpopulations in Peyer’s patches of mice and orients the immune response towards humoral immunity
Source: Gut Pathog. 2012 Dec 11;4:19. doi: 10.1186/1757-4749-4-19 (PMC3583793; doi:10.1186/1757-4749-4-19)
Supplement: Additional file 2 — Table S1. The population of commensal bacteria in cecal contents of mice. [file 1757-4749-4-19-S2.doc]

# Supplementary tables

## Table S1 - The population of commensal bacteria in cecal contents of mice

| **Microorganisms** | **Control**  (LgCFU/g *) | **Mild group**  (LgCFU/g *) | **Severe group**  (LgCFU/g *) |
| --- | --- | --- | --- |
| *Bifidobacterium*  *Lactobacillus*  *Enterococcus*  *Eubacterium*  *Streptococcus*  *Bacteroides*  *Enterobacter*  *Fusobacterium*  *Veilloella*  *Staphylococcus*  *Peptococcus*  *Aspergillus*  Yeast | 8.60±0.43  7.83±0.35  8.14±0.62  ND  8.94±0.39  7.26±0.95  7.34±1.25  7.04±0.55  8.45±0.11  7.67±0.24  4.38±0.48  2.86±0.36  6.62±0.22 | 5.16±0.09*  ND  ND  ND  5.41±0.03*  4.11±0.02*  3.40±0.13*  ND  5.48±0.13*  ND  3.20±1.06*  2.79±0.34  5.363±0.195* | ND**  ND  ND  ND  ND  ND  2.01±0.12*  ND  ND  ND  ND  3.14±0.16  ND |

All values are means ± SEM (n=5); * The bacterial colony-forming capacity was defined by the CFU formed by 1 g in logarithmic form; **ND means not detectable.

## Table S2 - Primers used for RT-PCR detection

|  | **Sequence of primers**  (5'-3') | **Product size** (bp) | **Tm**  (°C) |
| --- | --- | --- | --- |
| β-actin | F: TGGAATCCTGTGGCATCCATGAAAC  R: TAAAACGCAGCTCAGTAACAGTCCG | 348 | 60 |
| IL-2 | F: AGTCCACCACAGTTGCTGACTCA  R: TGTTCCCATCAAATGCTCGTTGGT | 283 | 57 |
| IFN-γ | F: TGAACGCTACACACTGCATCTTGG  R: TGACTCCTTTTCCGCTTCCTGAG | 399 | 62 |
| IL-4 | F: ATGGGTCTCAACCCCCAGCTAGTT  R: GCCCGAAAGAGTCTCTGCAGCTC | 237 | 61 |
| IL-10 | F: ACCTGGTAGAAGTGATGCCCCAGGCA  R:CTATGCAGTTGATGAAGATGTCAAA | 353 | 59 |
| TGF-β | F: TGACGTCACTGGAGTTGTACGG  R: GGTTCATGTCATGGATGGTGC | 170 | 61 |
